# Characterization of a New *qLTG3–1* Allele for Low-temperature Germinability in Rice from the Wild Species *Oryza rufipogon*

**DOI:** 10.1186/s12284-020-0370-2

**Published:** 2020-02-05

**Authors:** Kyu-Chan Shim, Sun Ha Kim, Hyun-Sook Lee, Cheryl Adeva, Yun-A Jeon, Ngoc Ha Luong, Woo-Jin Kim, Mirjalol Akhtamov, Yong-Jin Park, Sang-Nag Ahn

**Affiliations:** 1 Department of Agronomy, Chungnam National University, Daejeon, 34134 South Korea; 2 Department of Plant Resources, College of Industrial Science, Kongju National University, Yesan, 32439 South Korea

**Keywords:** Haplotype, Interspecific cross, Low-temperature germinability, Rice, Quantitative trait loci

## Abstract

**Background:**

Rice (*Oryza sativa* L.) is generally sensitive to low temperatures, and in production systems that use direct-seeding, low-temperature germinability (LTG) is a desired trait. Previously, the QTLs, *qLTG1* and *qLTG3,* that control LTG, were mapped using the BC_4_F_8_ population, which is a cross of Korean elite cultivar Hwaseong and *O. rufipogon* (IRGC 105491). We have characterized and analyzed the interaction between the two QTLs, by crossing TR20 that has *O. rufipogon* alleles at *qLTG1* and *qLTG3* in a Hwaseong background, with Hwaseong, to develop an F_2_ population.

**Results:**

The F_2_ plants with both *qLTG1* and *qLTG3* alleles from *O. rufipogon* showed higher LTG scores, than the plants with only *qLTG1* or *qLTG3*. No significant interaction between the *qLTG1* and *qLTG3* was observed, indicating that they may regulate LTG via different pathways. Based on its location, *qLTG3* appears to be allelic with *qLTG3–1*, a major QTL known to control LTG. To investigate the genetic differences between the two parents, that were controlling LTG, we compared their *qLTG3–1* sequences. In the coding region, three sequence variations leading to amino acid changes were identified between the Hwaseong and *O. rufipogon*. Of these, a non-synonymous substitution at the 62nd amino acid site, had not previously been reported. To understand the cause of the LTG variations between the parents, we genotyped three sequence variations of *qLTG3–1*, that were identified in 98 Asian cultivated rice accessions (*Oryza sativa* L.). The 98 accessions were classified into 5 haplotypes, based on three variations and a 71-bp deletion. Mean low-temperature germination rates were compared among the haplotypes, and haplotype 5 (*O. rufipogon*-type) showed a significantly higher germination rate than haplotype 2 (Nipponbare-type), and haplotype 3 (Italica Livorno-type).

**Conclusions:**

The *O. rufipogon qLTG3–1* allele can be utilized for the improvement of LTG in rice breeding programs. Nearly isogenic lines harboring both *qLTG1* and *qLTG3–1* alleles from *O. rufipogon,* showed higher LTG scores than the NILs with *qLTG1* or *qLTG3–1* alone, and the two QTLs regulate LTG via different pathways. To our knowledge, this is the first report to detect a new *qLTG3–1* allele and analyze the interaction of the two LTG QTLs in a nearly isogenic background.

## Background

Low-temperature germinability germinability (LTG) is one of the most important traits needed when using the direct-seeding rice cultivation method. Low-temperature-induced retardation of rice growth at the seed germination stage is a common problem in temperate and high-altitude tropical areas (Fujino and Sekiguchi, 2011). Improvement of LTG allows for high germination vigor and stable seedling establishment under low-temperature production environments, which leads to yield stability.

LTG is controlled by QTLs which have been detected using biparental populations and association analysis (Miura et al. 2001; Fujino et al. 2004; Jiang et al. 2006; Fujino et al. 2008; Ji et al. 2009; Nguyen et al. 2012; Fujino et al. 2015; Hyun et al. 2015; Wang et al. 2018). LTG QTLs have been identified on all 12 rice chromosomes, but many have relatively small effects, explaining less than 20% of the phenotypic variance. One exception is *qLTG3–1*, which was mapped in a population derived from a cross between Italica Livorno and Hayamasari, and conferred over 30% of the variation (Fujino et al. 2004). The *qLTG3*–*1* gene encodes a protein of unknown function, although it may be involved in tissue weakening (Fujino et al. 2008; Fujino and Sekiguchi 2011). Molecular markers linked to LTG have been employed in a marker-assisted selection program. For example, Li et al. (2019) developed a high LTG variety, DX71, by pyramiding 5 LTG QTLs from the cultivated rice variety ‘Xieqingzao B’, into a Dongxiang wild rice (DXWR) background.

Allelic variation in *qLTG3–1* identified in natural populations has been exploited, given the reportedly large effect of *qLTG3*–*1* on LTG variance. Functional nucleotide polymorphisms (FNPs) were originally identified during the cloning of in *qLTG3–1* including a 71-bp deletion and an amino acid substitution (A/T) (Fujino et al. 2008; Hori et al. 2010). Fujino and Sekiguchi (2011) further examined the sequence of *qLTG3–1* in 62 rice accessions from a core collection and detected 34 mutation events in its 1784-bp of 5′ upstream, coding, and 3′ downstream region*.* From these studies, Hayamasari (HY) variety had a 71-bp deletion compared to the Italica Livorno (IL), and an SNP (T/A) in the exon region was detected between IL and Nipponbare (NB). Hyun et al. (2015) screened a germplasm panel of 180 *japonica* rice accessions from temperate regions of Asia for the distribution of these three major alleles of *qLTG3–1* (IL, HY, and NB) based on their two polymorphisms. The results suggested that the IL, NB, and HY allele groups could be used to classify germplasm as LTG-tolerant, moderate, and sensitive, respectively. In contrast, Challam et al. (2013) evaluated a panel of 65 diverse Indian rice germplasm and did not distinguish between the IL and NB alleles of *qLTG3*–*1*. Considering that these studies mainly compared the effect of three major alleles on LTG, further studies to identify new beneficial *qLTG3*–*1* alleles are necessary for the improvement of low-temperature germinability.

In addition to identifying new *qLTG3–1* alleles that improve LTG, it is important to develop a better understanding of how the quantitative traits are regulated. QTL-QTL or gene-gene interactions need to be examined (Mackay 2014). Previous QTL studies mainly focused on identifying single locus genes, while studies on the interactions between genotypes at two or more QTL for LTG are limited (Miura et al. 2001; Fujino et al. 2004; Jiang et al. 2006; Fujino et al. 2008; Ji et al. 2009; Nguyen et al. 2012; Fujino et al. 2015).

Previously, we mapped two QTLs, *qLTG1* and *qLTG3*, that controlled low-temperature germinability, using a BC_4_F_8_ population derived from an interspecific cross between a Korean elite cultivar Hwaseong and *O. rufipogon* (IRGC 105491) (Nguyen et al. 2012; Shim et al. 2019).

In the present study, we characterized and analyzed the interaction between the two QTLs using an F_2_ population, derived from a cross between Hwaseong and TR20, a NIL with *O. rufipogon* alleles at *qLTG1* and *qLTG3* in the Hwaseong background. After validating the presence of the *qLTG3,* we compared the *qLTG3–1* sequences between the two parent lines and identified two sequence variations resulting in amino acid substitutions, as well as one 18-bp deletion in the coding region of *qLTG3–1* between Hwaseong and *O. rufipogon*. To determine which sequence variant among the three is associated with LTG variation, we genotyped the coding regions for *qLTG3–1* in 98 Asian rice accessions (*Oryza sativa* L.) from the KRICE_CORE (Kim et al. 2016). The 98 accessions were classified into 5 haplotypes based on the sequence variation in the coding region of *qLTG3–1.* Haplotype 5, which includes *O. rufipogon*, had a significantly higher mean low-temperature germination rate than the other groups, suggesting that the *O. rufipogon qLTG3–1* allele may be utilized for the improvement of low-temperature germinability in rice breeding programs.

## Materials and Methods

### Plant Materials

TR20 has four *O. rufipogon* chromosome segments on chromosomes 1, 3, 9, and 10, including the *qLTG1* and *qLTG3* regions (Additional file 1: Figure S1B). TR20 was crossed with Hwaseong for genetic analysis and three F_1_ plants were obtained. The F_1_ plants were self-pollinated to get F_2_ seeds and a total of 769 F_2_ plants were obtained. Among them, 224 F_2_ plants with enough F_3_ seeds for germination testing were selected for genotyping and phenotyping. These plants were genotyped using SSR markers on chromosomes 1, 3, 9, and 10 (McCouch et al. 2002). For *qLTG1*, the RM220, and CRM22 markers were used, whereas for the *qLTG3,* the RM60 and qLTG3–1_18D markers were used. To analyze the genetic interaction of *qLTG1* and *qLTG3,* 10 F_2_ plants each representing 4 groups (G11, G13, G31, and G33) were selected from the 224 F_2_ plants and tested for LTG (Additional file 1: Figure S1C). To determine the diversity of the *qLTG3–1* sequences (because *qLTG3* is allelic to *qLTG3–1*, the two QTLs are used interchangeably hereafter) among the rice accessions, we genotyped the coding regions of *qLTG3–1* in 98 Asian cultivated rice accessions (*Oryza sativa* L.) from the KRICE_CORE at Kongju National University (Kim et al. 2016); the accessions included 50 *temperate japonica*, 13 *tropical japonica*, 29 *indica*, 3 *aus*, 2 *admixture*, and 1 *aromatic* (Additional file 2: Table S1). The F_2_ population and parental lines were grown in the experimental paddy field at Chungnam National University, Daejeon, Korea, in the summer of 2017. The germinated seeds were sown on the 12th of April and 30-day-old seedlings were transplanted with 15 × 30 cm intervals. The KRICE_CORE set plants were grown in the experimental field at Chungcheongnam-do Agricultural Research and Extension Services (CNARES) in 2018.

### Evaluation of Low-Temperature Germinability

Germination tests were conducted as described by Nguyen et al. (2012) with minor modifications. Seeds of the F_2_ plants were collected 45 days after flowering. The harvested seeds were dried in a greenhouse for 2 weeks and stored at 55 °C for 3 days to break the seed dormancy. To confirm the breakage of seed dormancy, 15 seeds were germinated at an optimal germination temperature (30 °C). For low-temperature germination, 15 seeds were placed in a 6 cm petri dish with filter paper and filled with 5 ml of distilled water. Seeds were incubated in a growth chamber at 13 °C under dark conditions. Seed germination was considered to have occurred when the epiblast was broken, and the white embryo emerged (Additional file 1: Figure S2). Germinated seeds were counted and the germination rate (%) was calculated. The seed germination tests at 30 °C and 13 °C were carried out in duplicates and triplicates, respectively. All germination experiments were repeated two times in the same conditions. For the KRICE_CORE set, twenty seeds of each accession were incubated at an optimal germination temperature (30 °C) and at 13 °C with three replications, respectively. Rice accessions with an over 80% germination rate at the optimal germination temperature (30 °C), were used for LTG test.

### DNA Extraction, Genotype Analysis, and Sequencing

Fresh leaves from the F_2_ populations and parental lines, and the KRICE_CORE plants, were sampled and the DNA extractions were performed using the CTAB method (Causse et al. 1994). SSR markers were used to detect the *O. rufipogon* segments and marker information and genotype data are described in Yun et al. 2016. PCR reactions contained 10 ng of genomic DNA, 1 unit of *Taq* polymerase (Elpis), 2.5 uM each dNTP, 10 pmol of forward and reverse primers, and 10 x PCR buffer (10 mM Tris-HCl pH 8.3, 50 mM KCl, 1.5 mM MgCl_2_, 0.1% gelatin). PCR was performed as follows: 5 min of denaturation at 95 °C, 35 cycles of 3 steps; 98 °C for 20 s denaturation, 58 °C for 30 s annealing, 72 °C for 30 s extension, and 72 °C for 5 min for the final extension.

In the *qLTG3–1* coding region, three sequence variations were identified between Hwaseong and *O. rufipogon*. Two involved nucleotide substitutions (A/T and TTC/CGG) predicted to alter amino acids and one was an 18-bp deletion. To genotype the 98 rice accessions from the KRICE_CORE, primers pairs for three markers were designed (Additional file 2: Table S2). To detect the *qLTG3–1* A/T SNP at nucleotide position 50 in the exon, PCR products generated using the S103 marker were digested with *BseRI* (NEB), and separated on a 2 ~ 3% metaphor agarose gel. The marker qLTG3–1_TTC was designed to detect the GG**T TC**A/GG**C GG**A variant at nucleotide positions 183 to 185 (hereafter referred to as the TTC/CGG variant). The qLTG3–1_18D marker detected an 18-bp InDel starting at nucleotide position 127. PCR reactions consisted of 10 ng of genomic DNA, 10 pmol of forward and reverse primers, and EmeraldAmp® GT PCR Master Mix (Takara), and PCR was performed as follows: 5 min of denaturation at 98 °C, 35 cycles of 3 steps; 98 °C for 20 s denaturation, 60 °C for 30 s annealing, 72 °C for 30 s extension, and 72 °C for 5 min, for a final extension. The PCR products were separated on 2 ~ 3% metaphor agarose gels stained with StaySafe Nucleic Acid Gel Stain (RBC, Taiwan) or on 4% polyacrylamide denaturing gels, stained with Silver Staining Kit (Bioneer, Korea). Analysis of the *qLTG3–1* genomic sequences of Hwaseong and *O. rufipogon* was carried out by the SolGent sequencing service (SolGent Co. Ltd., Daejeon, Korea).

### Statistical Analysis and QTL Analysis

For one-way ANOVA and Tukey’s test, Minitab 16.2.4 software and R were used. The Student’s *t*-test was conducted using Microsoft Excel. QTL was declared by single-marker analysis when the phenotype was associated with the marker genotype at *P* < 0.01, in a one-way ANOVA.

## Results

### Comparison of the Seed Germination Rates between Parental Lines

**Fig. 1:**
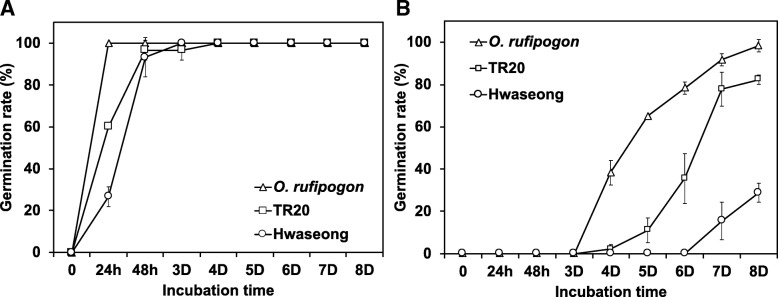
Germination rate of the parental lines (Hwaseong, *O. rufipogon*, and TR20) at (**a**) optimal temperature conditions (30 °C) and (**b**) low-temperature conditions (13 °C) for 8 days after incubation. Triangle, square, and circle indicate means of germination rate for *O. rufipogon*, TR20, and Hwaseong, respectively. Error bars indicate standard error

The germination rates of the parental lines (Hwaseong, *O. rufipogon,* and TR20) were compared at the optimal (30 °C) and low temperature (13 °C) conditions (Fig. 1). *O. rufipogon* had a germination rate of about 100% 1 day after incubation (DAI), while TR20 and Hwaseong had germination rates of nearly 100% after 2 DAI. TR20 had a slightly higher germination rate than the Hwaseong at 30 °C after 1 DAI. This indicated that the seeds of the parental lines have normal germination capabilities at the optimal condition (30 °C). In the low-temperature condition, *O. rufipogon* showed the highest germination rate at 4 DAI and reached 100% after 8 DAI. The germination rate of the TR20 was significantly higher than that of the Hwaseong, as the TR20 began to germinate at 4 DAI, while the Hwaseong began to germinate at 7 DAI. The differences in the germination rates were largest after 7 DAI, between the Hwaseong and TR20.

### Validation and Evaluation of *qLTG1* and *qLTG3*

We used the low-temperature germination rates of the F_2_ population at 7 DAI for the QTL analysis, based on the previous findings that the largest differences between the parental lines (TR20 and Hwaseong) were at 7 DAI. We found that 224 F_2_ plants showed nearly normal distributions of low-temperature germination at 7 DAI (skewness = − 0.05) whereas the germination rate at 6 DAI showed a right skewed distribution (skewness = 0.77) (Additional file 1: Figure S3). QTL analysis detected two significant QTLs on chromosomes 1 and 3. No QTLs were observed on chromosomes 9 and 10 (*P* = 0.796 for RM1533 on Chr. 9 and *P* = 0.463 for Chr10_InDel4 on Chr. 10). *qLTG1* was located between RM220 and CRM22 on chromosome 1 and explained 13.1% of the total phenotypic variation in the F_2_ population, and the *O. rufipogon* allele increased LTG (Table 1). *qLTG3* was detected between RM60 and qLTG3–1_18D on chromosome 3 and explained 39.9% of the phenotypic variation (Table 1). The interaction between the two QTLs was not significant (*F* = 0.23, 0.6 < *P* < 0.7). Gene action of the two QTLs, *qLTG1* and *qLTG3*, was determined using the F_2_ plants, segregating only for the *qLTG1* or *qLTG3* region, respectively (Additional file 1: Figure S4). The additive effect (*a*) of the *O. rufipogon* allele at *qLTG1* were 8.5% with a dominance effect of − 4.8% (*d*), indicating that the *O. rufipogon* allele at *qLTG1* is partially recessive, in regulating LTG (Additional file 1: Figure S4A). The additive effect and dominance effect of the *O. rufipogon* allele at *qLTG3* were 15.7 and 16.3%, respectively. The degree of dominance (*d/a*) was 1.0, indicating that the *O. rufipogon* allele behaves in a dominant manner (Additional file 1: Figure S4B).

**Table 1:** QTL analysis for low-temperature germinability in the F_2_ population

Trait^a^	QTL	Chr.	Marker	*P*-value	R^2b^ (%)
7 DAI	*qLTG1*	1	RM220 - CRM22	0.000	13.1
	*qLTG3*	3	RM60 - qLTG3–1_18D	0.000	39.9
Interaction^c^			RM220/RM60	0.629	–
Total^d^				–	47.3

^a^DAI: days after incubation, ^b^ R^2^: Coefficient of determination, ^c^ Interaction between *qLTG1* and *qLTG3*, ^d^ Total phenotypic variance was determined by regression analysis

### Interaction of the Two LTG QTLs

**Fig. 2:**
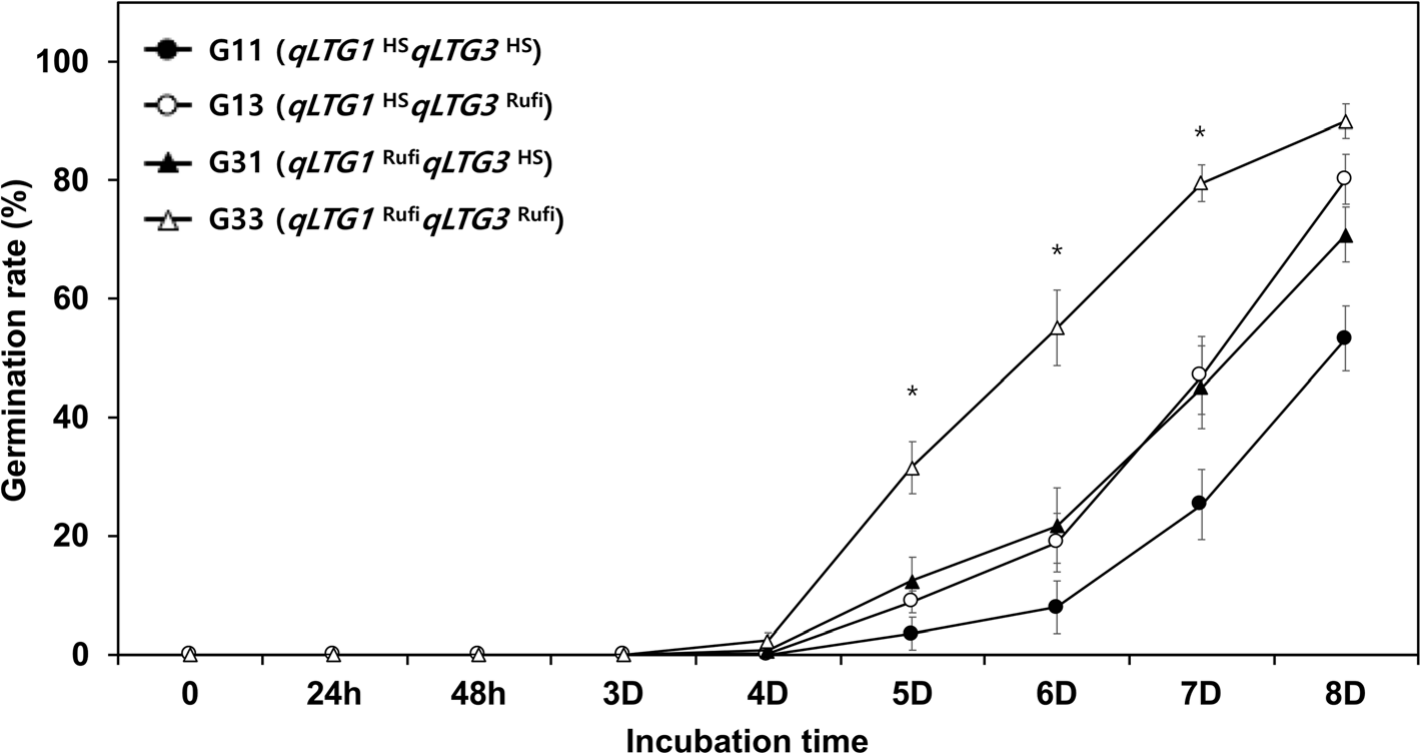
Comparison of the germination rates of the four genotype groups at 13 °C. *qLTG1*^HS^ and *qLTG1*^Rufi^ indicate homozygosity for Hwaseong and *O. rufipogon* genotype at the *qLTG1* locus, respectively. *qLTG3*^HS^ and *qLTG3*^Rufi^ indicate homozygosity for Hwaseong and *O. rufipogon* at the *qLTG3* locus. * indicates a significant difference of *P* < 0.05 based on ANOVA. Error bars indicate standard error

Based on the genotypes at the loci *qLTG1* and *qLTG3*, F_2_ plants representing the four genotype groups (G11, G13, G31, and G33) were selected (Additional file 1: Figure S1). The LTG of the four genotype groups was compared at 13 °C (Fig. 2). The G33 plants harboring both *O. rufipogon* alleles at *qLTG1* and *qLTG3* showed significantly higher LTG than the other 3 groups at 5, 6, and 7 DAI. The germination rate of the G33 plants at 7 DAI was about 80%, while the germination rates of the other genotype groups remained under 50%. Both the G13 and G31 showed higher germination rates after 6 DAI, compared with the G11. These results indicated that the *qLTG1* and *qLTG3 O. rufipogon* alleles increased the LTG in an additive manner and pyramiding the *O. rufipogon* with the *qLTG1* and *qLTG3* alleles in a *japonica* rice background would be an effective method for enhancing LTG.

### Sequence Comparisons of *qLTG3*

**Fig. 3:**
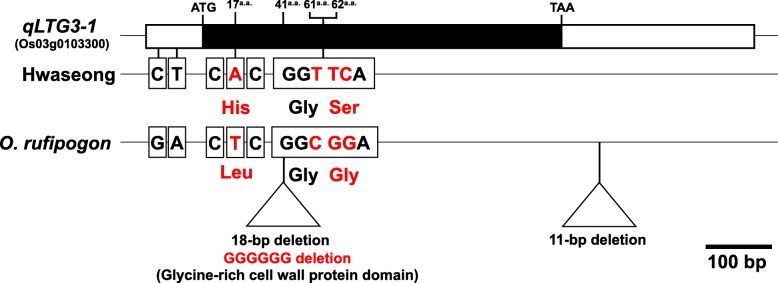
Sequence comparison of the *qLTG3–1* gene between the Hwaseong and *O. rufipogon.* The black box indicates an exon with the position of the amino acid sequence from the start

Based on the physical location and the major effects on LTG, *qLTG3* appears to be allelic with the previously cloned *qLTG3–1* (Os03g0103300) gene (Fujino et al. 2008). We compared the *qLTG3–1* sequences between the Hwaseong and *O. rufipogon* varieties to identify the differences in LTG between the two parents. A total of six SNPs and two InDels were detected in the 5` UTR, exon, and 3` UTR regions (Fig. 3). Three sequence differences were observed in the coding region. An A/T SNP located in the exon region at nucleotide position 50, predicted to encode an amino acid difference from Leu (*O. rufipogon*) to His (Hwaseong), and an 18-bp deletion starting from nucleotide position 127 corresponding to the deletion of six glycines (G) starting at the 41st amino acid, were detected in *O. rufipogon*. In addition, a variant of TTC/CGG was observed from nucleotide position 183 to 185, encoding Ser in Hwaseong and Gly in *O. rufipogon*. These nucleotide differences between the Hwaseong and *O. rufipogon* might be responsible for the variations in LTG. Of these sequence variations, the non-synonymous substitution at the 62nd amino acid site has not previously been reported. According to previous haplotype studies, Hwaseong has the same haplotype as Nipponbare (Allele 5 of Allele group I), whereas *O. rufipogon* displayed a unique haplotype different from the other 10 haplotype groups (Fujino and Sekiguchi 2011). While the two nucleotide substitution variants (A/T and TTC/CGG) and the 18-bp deletion have been identified between Hwaseong and *O. rufipogon*, which variation is responsible for the differences in LTG between the two parental lines is not clear. To clarify which variation is associated with LTG, additional experiments including transgenic approach are necessary.

### Development of *qLTG3* Allele Specific Markers

To genotype the sequence variations, *qLTG3–1* allele specific markers were developed (Additional file 2: Table S2). The CAPS marker S103 (*Bse*RI) detects the A/T SNP at nucleotide position 50 and a 71-bp deletion (Fujino et al. 2008). Three marker sets were designed to detect the 18-bp InDel and TTC/CGG variants (Additional file 1: Figure S5). qLTG3–1_18D was designed to detect the presence/absence of the 18-bp deletion. qLTG3–1_TTC, a dominant marker containing a mismatch near the 3`-terminus can specifically amplify the TTC sequence whereas qLTG3–1_CGG can amplify the genomic region with the CGG sequence. S103 produced a single PCR band in Hwaseong and *O. rufipogon* (Additional file 1: Figure S5). When this band was digested with *Bse*RI, it produced the polymorphism between Hwaseong and *O. rufipogon* due to the A/T SNP at nucleotide position 50 (Additional file 1: Figure S5). qLTG3–1_18D marker produced 208-bp and 190-bp size bands in Hwaseong and *O. rufipogon*, respectively on 3% a metaphor agarose gel. For qLTG3–1_TTC, a 166-bp PCR amplicon was produced in Hwaseong, whereas no band was detected in *O. rufipogon* (Additional file 1: Figure S5). Instead, *O. rufipogon* showed a 166-bp product from the PCR with the qLTG3–1_CGG marker while no target PCR amplicon was detected from Hwaseong. The three newly developed markers and S103 (*Bes*RI), successfully detected polymorphisms between the two parents.

### Genotyping and Haplotype Analysis of *qLTG3*

**Fig. 4:**
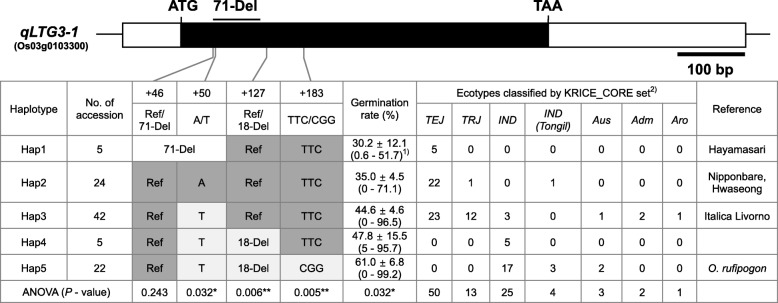
Haplotype analysis of the *qLTG3–1* using the 98 rice accessions from the KRICE_CORE set. The germination rate was represented as mean ± standard error and measured 6 days after incubation at 13 °C. ^1)^ Values in parenthesis are minimum and maximum germination rates of each haplotype. ^2)^ *TEJ* = *temperate japonica*; *TRJ* = *tropical japonica*; *IND* = *indica*; *IND (Tongil)* = *indica Tongil*; Aus = *aus*; *Adm* = *admixture*; *Aro* = *aromatic*

To determine which of the three sequence variations in the *qLTG3–1* exon region is associated with the LTG variation, genotyping and haplotype analysis were performed for 98 accessions from the KRICE_CORE set (Additional file 2: Table S1). With the S103 marker, 5 (Hap1) of the 98 accessions showed a 71-bp deletion band (Fig. 4, Additional file 1: Figure S6A). The S103 PCR product, when treated with *Bse*RI, revealed that 24 (Hap2) and 69 of the rice accessions had A and T genotypes, respectively (Fig. 4). With the qLTG3–1_18D, three different bands including 2 parental bands were amplified. Five accessions (Hap4) displayed a PCR band that was 18 and 36-bp bigger than the Hwaseong and *O. rufipogon* alleles, respectively (Fig. 3, Additional file 1: Figure S5, S6). Sequence analysis revealed that these five accessions have the target 18-bp deletion and a 36-bp insertion starting from nucleotide position 190. This result was consistent with the finding by Fujino and Sekiguchi (2011) (Additional file 1: Figure S5). With regard to the 18-bp deletion in the exon, 27 accessions (Hap4 and 5) had deletions, whereas the remaining 71 accessions had Hwaseong (Reference) type (Hap1, 2 and 3). Two dominant markers, qLTG3–1_TTC and qLTG3–1_CGG, share the same forward primer and their reverse primers were designed to anneal specifically to their TTC and CGG sites, respectively. The two primers for qLTG3–1_TTC and qLTG3–1_CGG failed to amplify the target fragments (166-bp) in ten accessions (Hap1 and 4), because the forward primers of qLTG3–1_TTC and qLTG3–1_CGG were designed to include a 71-bp deletion (Hap1) and 36-bp insertion (Hap4) includes the reverse primers of qLTG3–1_TTC and qLTG3–1_CGG (Additional file 1: Figure S6). For qLTG3–1_CGG, Hap4 rice accessions had larger amplicons than the 166-bp target band, as the qLTG3–1_CGG reverse primer (3`-direction) annealed to the end of the 36-bp insertion site (Additional file 1: Figure S5, S6B). Sequence analysis revealed that the ten accessions have the TTC genotype (Additional file 1: Figure S6B). Taken together, 76 (Hap1, 2, 3, and 4) and 22 (Hap5) accessions showed TTC and CGG genotypes, respectively.

Based on genotypes across the *qLTG3–1* coding region, we classified 98 accessions into 5 haplotypes (Fig. 4). Five *temperate japonica* accessions (5.1%) with a 71-bp deletion belonged to Hap1 (Hayamasari type). Hap2 (24.5%) was mainly composed of *temperate japonica* including Hwaseong and Nipponbare except for one *Tongil* type ‘Milyang50’ which was derived from a cross between *japonica* and *indica.* Hap3 was the most prevalent type and consisted of *temperate* and *tropical japonica* accessions with the Italica Livorno allele. Five *indica* accessions (5.1%) belonged to Hap4 and all accessions had a 36-bp insertion at nucleotide position 190 (Additional file 1: Figure S6). *O. rufipogon* was classified as Hap5, along with 17 *indica,* 3 *Tongil,* and 2 *aus* (22.4%). When the three sequence variants (C/G, T/A, and 11-bp deletion) in 5′ and 3′ UTR region between Hwaseong and *O. rufipogon* were compared in the rice core accessions, the two SNP G and A, and the 11-bp deletion showed complete cosegregation with the Hap4- and Hap5-specific 18-bp deletion genotype. Fujino et al. (2011) also reported that C/G and T/A SNP on 5′ UTR region showed complete linkage with 18-bp deletion on 3′ UTR region. Therefore, three sequence variants (C/G, T/A, and 11-bp deletion) would be informative to select for Hap4 and Hap5 accessions.

### Comparison of LTG among the Five *qLTG3–1* Haplotypes

**Fig. 5:**
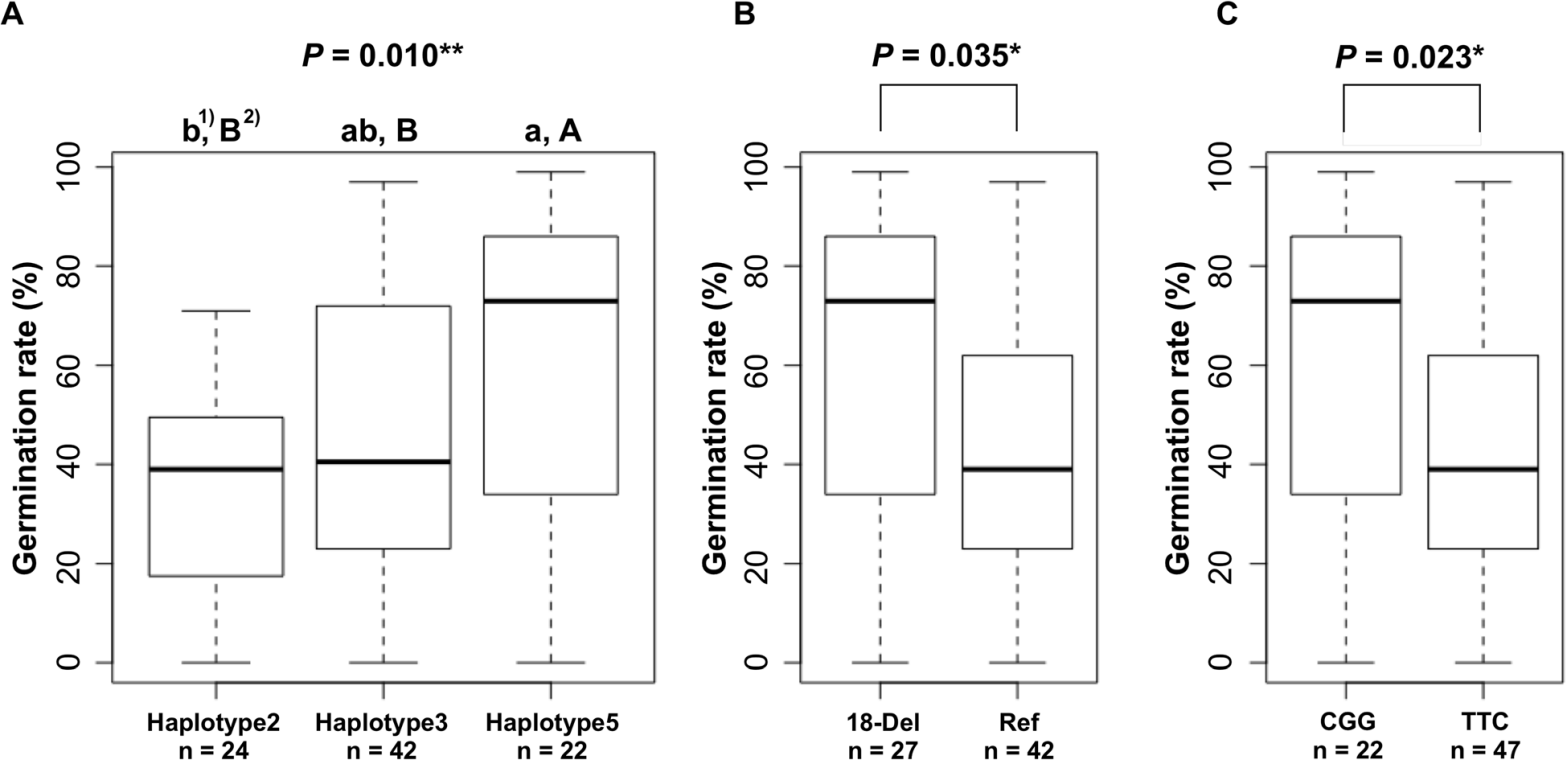
Comparison of the germination rates (**a**) between haplotypes 2, 3, and 5, and (**b**) between the accessions with the 18-bp deletion and the reference type, and (**c**) between the CGG type and the TTC type. * and ** indicate significant differences of *P* < 0.05 and 0.01, respectively, as determined from ANOVA and *t*-tests. ^1),2)^ The same letter is not significantly different among the three haplotypes at *P* = 0.05^1)^ and *P* = 0.10^2)^ based on Tukey’s test, respectively

To identify the cause of variation in LTG, LTG was evaluated in the 98 accessions from the KRICE_CORE set (Additional file 1: Figure S5). The germination rate at 6 DAI at 13 °C, was used for analysis because the germination rate showed a normal distribution at 6 DAI (skewness = 0.10, data not shown). Comparison of the LTG among the 98 accessions by ecotype, indicated the median of germination rate in the *indica* and *Tongil* rice varieties was higher than that for the *temperate* and *tropical japonica*. Furthermore, the *admixture*, *aromatic*, and *aus* types showed lower germination rates than the *japonica* rice (data not shown). When the mean germination rates were compared by haplotype group, Hap1 displayed the lowest germination rate of 30.2% at 6 DAI, followed by Hap2 at 35.0% under low-temperature conditions (Fig. 4). The germination rate of Hap3 was 44.6%, whereas for Hap4 and Hap5 it was 47.8 and 61.0%, respectively. The germination rate of the Hap5 (61.0%) which included *O. rufipogon* was significantly higher than that of Hap2 (35.0%) with Hwaseong and Nipponbare (Fig. 5). In addition, the accessions in the Hap5 group (61.0%) had a significantly higher low-temperature germination rate than those in Hap3 (44.6%) which included Italica Livorno. These results suggest that the *O. rufipogon* allele has a greater positive effect on LTG than the Italica Livorno allele.

SNP genotyping was used to identify which sequence differences among the three were associated with variations in the LTG (Fig. 4). Significant differences were found in the LTG of the two groups differing at the A/T SNP; accessions with T showed significantly higher LTG than the accessions with A (*P* = 0.032). The germination ratios of accessions in Hap 4 and Hap5, which both harbored the 18-bp deletion, were significantly higher than the accessions with the reference sequence (*P* = 0.006). Furthermore, the CGG allele type had a higher germination rate than the TTC allele type. Among the three variations, TTC/CGG showed the most significant *P*-value among the haplotypes (*P* = 0.005).

Previously, the A/T SNP was shown to be a functional nucleotide polymorphic site with the T allele conferring an increased LTG compared to the A allele (Hori et al. 2010). When the three haplotypes, Hap2, Hap3, and Hap5, were compared for LTG (Hap1 and Hap4 were excluded due to small sample sizes), Hap2 and Hap3 were not significantly different in LTG despite Hap2 and Hap3 having different alleles at the A/T site (Fig. 5a). Hap5 plants showed significantly higher LTG than the Hap2 and Hap3 plants, based on the Tukey’s test (*P* < 0.07), and these results suggest that the A/T SNP would not be informative as a functional marker for all haplotypes.

To determine whether the 18-bp deletion affected LTG, we compared the LTG among three haplotypes (Hap3, 4, and 5) which have the T allele at position 50. The LTG of the Hap4 and Hap5 accessions, which harbor the 18-bp deletion, was significantly higher than the reference types (Hap3) (Fig. 5b). Also, the accessions with the CGG allele showed significantly higher LTG than those carrying the TTC allele. These results suggested that not only the 18-bp deletion, but also the TTC/CGG variation were associated with differences in the LTG of the rice accessions.

## Discussion

LTG is one of the essential traits for direct-seeding cultivation in rice. In this study, two QTLs, *qLTG1* and *qLTG3,* were validated using an F_2_ population derived from a cross between Hwaseong and TR20, which harbors four *O. rufipogon* chromosome segments in the Hwaseong genetic background. Based on its similar location and the major effect on the LTG, *qLTG3* appears to be allelic to *qLTG3–1* (Fujino et al. 2008). To determine the genetic basis of the differences in the LTG between the two parental lines, the sequences of the two parents at *qLTG3–1* were analyzed. Three differences were found in the coding region of *qLTG3–1* between Hwaseong and *O. rufipogon*. Two of the variations, an A/T SNP at position 50 and an 18-bp deletion, have previously been identified (Hori et al. 2010; Fujino and Sekiguchi, 2011). However, the non-synonymous variation TTC/CGG has not previously been reported, suggesting that *O. rufipogon* has a new and trait-improving allele at *qLTG3–1*. As few attempts have been made to utilize wild rice resources to improve LTG, this study clearly highlights and supports the importance of utilizing natural variation for functional and breeding research of LTG (Kovach and McCouch, 2008; Alonso-Blanco et al. 2009). Although the gene corresponding to *qLTG1* remains to be identified, genetic analysis of the F_2_ population suggested the recessive nature of the *O. rufipogon* allele of *qLTG1* since the heterozygous plants were similar in LTG to the Hwaseong homozygous plants, but lower than the *O. rufipogon* homozygous plants with a degree of dominance of − 0.6. The *O. rufipogon* allele at *qLTG3* was dominant in regulating the LTG, with a degree of dominance of 1.0. This result is similar with an earlier study showing that the Italica Livorno allele in *qLTG3–1* (Os03g0103300) was dominant over the Hayamasari allele (Fujino et al. 2008).

The interaction between the two QTLs, *qLTG1* and *qLTG3,* was examined using 4 genotype groups (Fig. 2). The plants which harbor the two QTLs from *O. rufipogon* showed the highest germination rates at 13 °C from the 4 groups, and the two QTLs cumulatively explained 47.3% of the phenotypic variance in the LTG. These results imply that two QTLs control the LTG in an additive manner. Pyramiding these two QTLs from the *O. rufipogon* into cultivated rice would be valuable for breeding programs interested in enhancing LTG for direct-seeding production systems. It is also noteworthy that the plants with two *O. rufipogon* alleles at *qLTG1* and *qLTG3* showed lower LTG than the *O. rufipogon*, the donor parent at 5–7 DAI, indicating the presence of additional QTL for LTG in *O. rufipogon*. Further experiments are underway to detect and characterize these unknown QTLs in *O. rufipogon*.

For the efficient utilization of the favourable alleles of *qLTG3–1*, the identification of new *qLTG3–1* alleles and examination of their distribution are useful for rice breeding programs. To accomplish this goal, DNA sequences of the coding region in *qLTG3–1* in the 98 rice accessions were analyzed (Kim et al. 2016). The DNA primers that were used to differentiate between the two variations, an 18-bp deletion and the TTC/CGG of Hwaseong and *O. rufipogon,* were utilized to assay the 98 rice accessions along with the S103 marker which detects alleles containing a 71-bp deletion and A/T functional nucleotide polymorphism (Fujino et al. 2008).

Based on the genotype data across the *qLTG3–1* coding region, the 98 accessions were classified into 5 distinct haplotypes. The haplotype classifications in this study were similar to those reported by Fujino and Sekiguchi (2011). Five *japonica* rice accessions were included in Hap1 (Hayamasari type) and these include four collections from Korea (Suwon301, PyungBook 3, IRI336, and Jeju collection) and one from Japan (Gou 405). Hap2 (Hwaseong and Nipponbare type) was found in 23 *temperate* and *tropical japonica* accessions and one *Tongil-*type accession, Milyang50. Milyang50 developed in Korea is the progeny of Milyang23, which carries the Nipponbare genotype at *qLTG3–1* and this result is consistent with the findings of Fujino and Sekiguchi (2011).

Hap3 (Italica Livorno type) consisted of various ecotypes (35 *temperate* and *tropical japonica,* 3 *indica,* 1 *aus,* and 1 *aromatic*, 42.5%). Five *indica* accessions (SaDuCho, Sun, BaekGakWhara, Hatadani, and Qua 77 Wuan Dau) with a 36-bp insertion at position 190 were included in Hap4. Hap4 corresponded to Allele group III (Allele 9) classified by Fujino and Sekiguchi (2011), and Allele 9 was comprised of four *indica* rice accessions (Fujino and Sekiguchi, 2011). These two independent studies suggest that the 36-bp insertion might be an *indica* specific mutation event.

Hap5 (*O. rufipogon* type) includes 17 *indica*, 3 *Tongil*, and 2 *aus* rice accessions. The three *Tongil* rice cultivars in Hap5 were Yeongpung Byeo, Suwon 148, and Suwon 255. Although the Yeongpung Byeo is the progeny of a Milyang23/IR2061 cross, the Milyang23 allele at *qLTG3–1* was not selected in the breeding program, implying that the LTG was not the major target trait in that breeding program. This hypothesis will be tested using more breeding lines and germplasm developed in Korea. Although this haplotype showed the highest LTG, two rice accessions (Tchampa and Mala collected from Iran and Bangladesh, respectively) showed very low LTG. This finding suggests that these accessions might possess relatively more negative genes that lower LTG and additional markers linked to LTG should be tested in evaluating the diverse germplasm. The TTC/CGG variation was newly identified in this study, and Hap5 specifically has the CGG allele. Fujino and Sekiguchi (2011) reported that Allele group II (Allele 6–8) has two sequence variations; a 9-bp deletion and a 9-bp insertion at positions 181 and 190, respectively. As the TTC/CGG variation at position 183 overlapped with a previously reported 9-bp deletion at position 181, it would not have been possible to detect the TTC/CGG in the study by Fujino and Sekiguchi (2011).

When mean germination rates among the three haplotypes (Hap2, Hap3, and Hap5) were compared, Hap5 showed significantly higher LTG than Hap2 and Hap3. Among the accessions with the T SNP allele at position 50, the 18-bp deletion and CGG allele showed higher LTG than the reference and TCC allele types, respectively. Interestingly, the 18-bp deletion and CGG allele, which are present in the *O. rufipogon qLTG3–1* gene, result in a significant reduction in the number of glycines (seven in total) in the glycine-rich cell wall protein domain (amino acids 1–100) of the qLTG3–1 protein. Various glycine-rich proteins (GRPs) have been reported from different organisms and their glycine contents showed a large amount of variation (Ringli et al. 2001). In addition, GRPs have biochemical characteristics which contribute to the strengthening of biological structures or which allow the formation of very tensile fibers (Ringli et al. 2001). It would be reasonable to expect that the reduced number of glycines would affect the function of the protein and could possibly cause tissue weakening, leading to the better LTG observed with the *O. rufipogon* allele compared to that of the Italica Livorno (Hap3). It is noteworthy that LTG was not significantly different between Hap1 with 71-bp deletion in *qLTG3–1* and Hap2 and this result is not consistent with the previous report that the 71-bp deletion in *qLTG3–1* causes a frameshift mutation, thereby leading to a decrease in LTG (Fujino et al. 2008; Hyun et al. 2015). This might be partly due to possible interactions among genes affecting LTG, and different sample size (five in Hap1 vs 24 Hap2). In Hap1, RWG-079 and RWG-092 showed about 2 and 1% of LTG, while RWG-004, RWG-050, and RWG-079 showed from 40 to 56% of LTG at 6 DAI (Additional file 2: Table S1). The large phenotypic variation and small sample size of Hap1 may have resulted in a lack of significant difference between Hap 1 and Hap 2.

In this study, a new *qLTG3–1* allele for LTG from *O. rufipogon* was characterized. The 18-bp deletion and TTC/CGG in the exon region are predicted to change the amino acid in the glycine-rich cell wall protein domain and these changes might be responsible for the phenotypic variations in LTG. Although wild-QTL alleles that are favorable for some traits may be associated with deleterious effects on other traits (Xiao et al. 1998), the *qLTG3–1* region in the *O. rufipogon* was not associated with negative effects on agronomic traits and grain quality traits in *japonica* backgroung (Yun et al. 2016). Functional markers for LTG developed in this study could be applied in screening for high LTG potentials in rice germplasm collections. The introgression of the desirable QTLs from wild rice is a promising approach to be utilized in breeding new abiotic tolerant lines.

## Conclusions

We demonstrated that the *O. rufipogon qLTG3–1* allele can be utilized for the improvement of LTG in rice breeding programs, supporting the importance of utilizing natural variation for functional and breeding researches in LTG. Nearly isogenic lines harboring both *qLTG1* and *qLTG3–1* alleles from *O. rufipogon* showed better LTG than NILs with *qLTG1* or *qLTG3–1* alone implying that two QTLs regulate LTG via different pathways. To our knowledge, this is the first report to detect a new *qLTG3–1* allele and analyze the interaction of two LTG QTLs in nearly isogenic backgrounds. Functional markers for *qLTG3–1* developed in this study may be applied for screening high LTG germplasms in rice collections.

## Supplementary information

**Additional file 1: Figure S1.** Graphical genotypes of the parental lines (A) Hwaseong, (B) TR20, and (C) four genotype groups from the F_2_ plants. White and black bars indicate the Hwaseong and *O. rufipogon* chromosome segments, respectively. **Figure S2.** Determination of seed germination of (A) *O. rufipogon*, (B) Hwaseong, and (C) TR20 on filter papers (60 mm) in the petri dishes (60 mm) at 13 °C for 4–8 days after incubation. Arrowheads indicate the germinated embryo. **Figure S3.** Frequency distribution of LTG at 6 days (A) and 7 days (B) after incubation of the 224 F_2_ plants, respectively. Triangles and horizontal lines denote mean germination rates and standard error of the parental lines, respectively. **Figure S4.** Comparison of the germination rates in plants that are segregating at (A) *qLTG1* and (B) *qLTG3* regions without other LTG QTL. RR, RH, and HH, mean *O. rufipogon* homozygous, heterozygous, and Hwaseong homozygous with the number of F_2_ individuals, respectively. Additive effect (*a*) of the *O. rufipogon* allele, dominance effect of the *O. rufipogon* allele (*d*), and degree of dominance (*d/a*) were indicated in each figure. The same letter above box is not significantly different between the genotypes at *P* = 0.05 based on Tukey’s test. **Figure S5.** PCR amplicons of the markers used for genotyping parental lines and rice accessions for haplotype analysis. HS: Hwaseong, Rufi: *O. rufipogon*, #: RWG accession number, and M: 100-bp size marker. **Figure S6.** Sequence comparisons of the Ref (Nipponbare), HS (Hwaseong), Rufi (*O. rufipogon*) and (A) five rice accessions (RWG4, RWG50, RWG79, RWG87, RWG92) with a 71-bp deletion and (B) five rice accessions (RWG21, RWG22, RWG47, RWG54, RWG70) with a 36-bp insertion. Sequence and location of 3 markers are shown with an A/T SNP, 18-bp and 71-bp deletions, TTC/CGG, and 36-bp insertion variant site.

**Additional file 2: Table S1.** List of KRICE_CORE accessions used for low-temperature germinability test. **Table S2.** Primer list used in this study.

## Data Availability

All data supporting the conclusions described here are provided in tables and figures.

## Abbreviations

CAPS: Cleaved amplified polymorphic sequence
InDel: Insertion and deletion
LTG: Low-temperature germinability
SNP: Single nucleotide polymorphism
SSR: Simple sequence repeat
