# Supplementary material for: Characterization of a New qLTG3–1 Allele for Low-temperature Germinability in Rice from the Wild Species Oryza rufipogon
Source: Rice (N Y). 2020 Feb 5;13:10. doi: 10.1186/s12284-020-0370-2 (PMC7002630; doi:10.1186/s12284-020-0370-2)
Supplement: Supplementary file 1 — Additional file 1: Figure S1. Graphical genotypes of the parental lines (A) Hwaseong, (B) TR20, and (C) four genotype groups from the F2 plants. White and black bars indicate the Hwaseong and O. rufipogon chromosome segments, respectively. Figure S2. Determination of seed germination of (A) O. rufipogon, (B) Hwaseong, and (C) TR20 on filter papers (60 mm) in the petri dishes (60 mm) at 13 °C for 4–8 days after incubation. Arrowheads indicate the germinated embryo. Figure S3. Frequency distribution of LTG at 6 days (A) and 7 days (B) after incubation of the 224 F2 plants, respectively. Triangles and horizontal lines denote mean germination rates and standard error of the parental lines, respectively. Figure S4. Comparison of the germination rates in plants that are segregating at (A) qLTG1 and (B) qLTG3 regions without other LTG QTL. RR, RH, and HH, mean O. rufipogon homozygous, heterozygous, and Hwaseong homozygous with the number of F2 individuals, respectively. Additive effect (a) of the O. rufipogon allele, dominance effect of the O. rufipogon allele (d), and degree of dominance (d/a) were indicated in each figure. The same letter above box is not significantly different between the genotypes at P = 0.05 based on Tukey’s test. Figure S5. PCR amplicons of the markers used for genotyping parental lines and rice accessions for haplotype analysis. HS: Hwaseong, Rufi: O. rufipogon, #: RWG accession number, and M: 100-bp size marker. Figure S6. Sequence comparisons of the Ref (Nipponbare), HS (Hwaseong), Rufi (O. rufipogon) and (A) five rice accessions (RWG4, RWG50, RWG79, RWG87, RWG92) with a 71-bp deletion and (B) five rice accessions (RWG21, RWG22, RWG47, RWG54, RWG70) with a 36-bp insertion. Sequence and location of 3 markers are shown with an A/T SNP, 18-bp and 71-bp deletions, TTC/CGG, and 36-bp insertion variant site. [file 12284_2020_370_MOESM1_ESM.pdf]

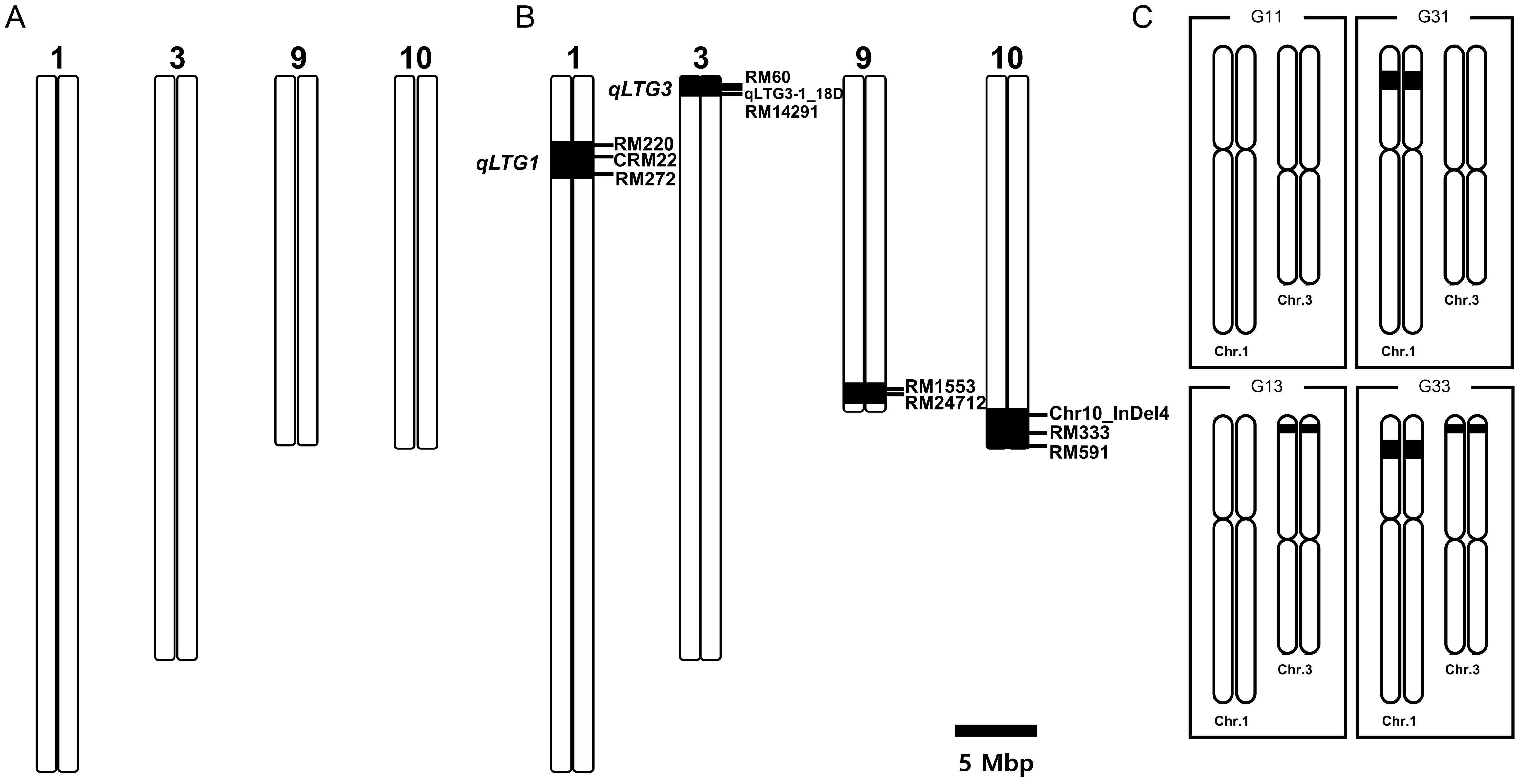

Figure S1. Graphical genotypes of the parental lines (A) Hwaseong, (B) TR20, and (C) four genotype groups from the F_2 plants. White and black bars indicate the Hwaseong and *O. rufipogon* chromosome segments, respectively.

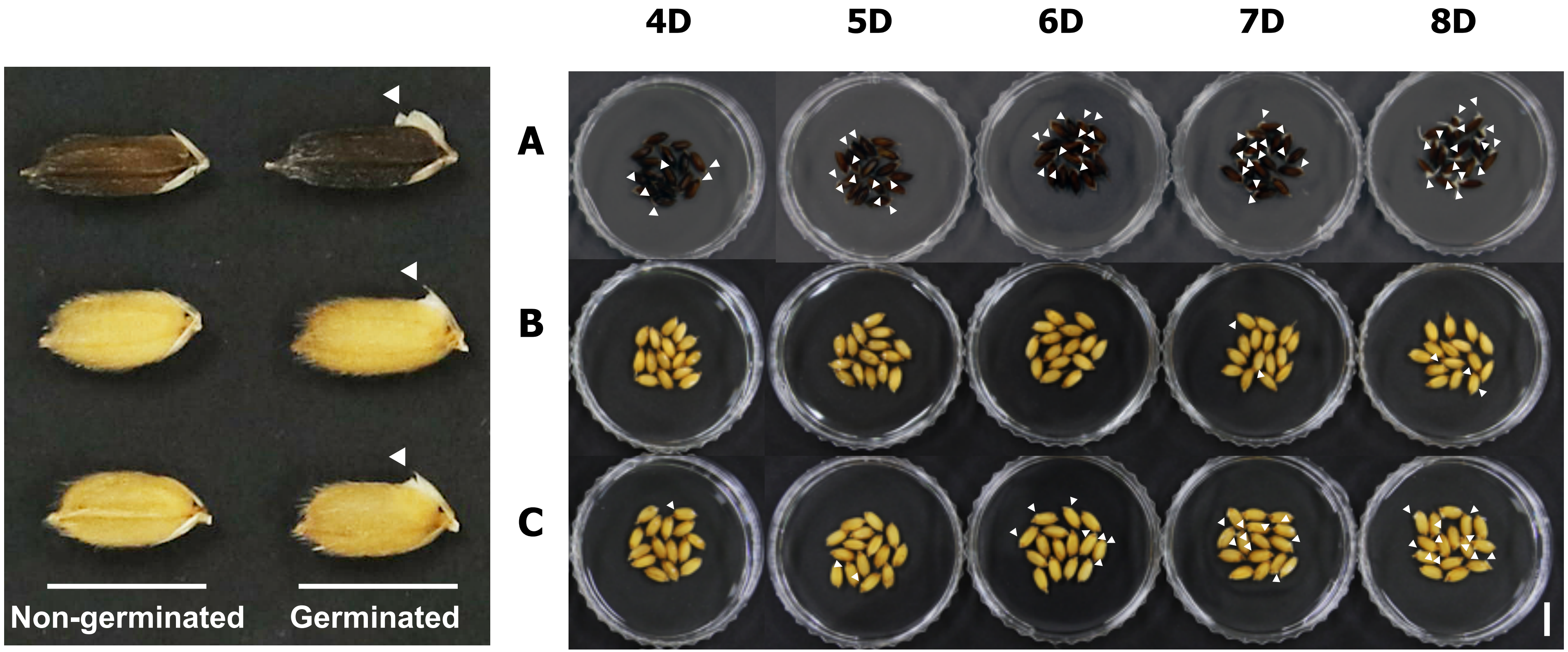

Figure S2. Determination of seed germination of (A) *O. rufipogon*, (B) Hwaseong, and (C) TR20 on filter papers (60 mm) in the petri dishes (60 mm) at 13 ℃ for 4 - 8 days after incubation. Arrowheads indicate the germinated embryo.

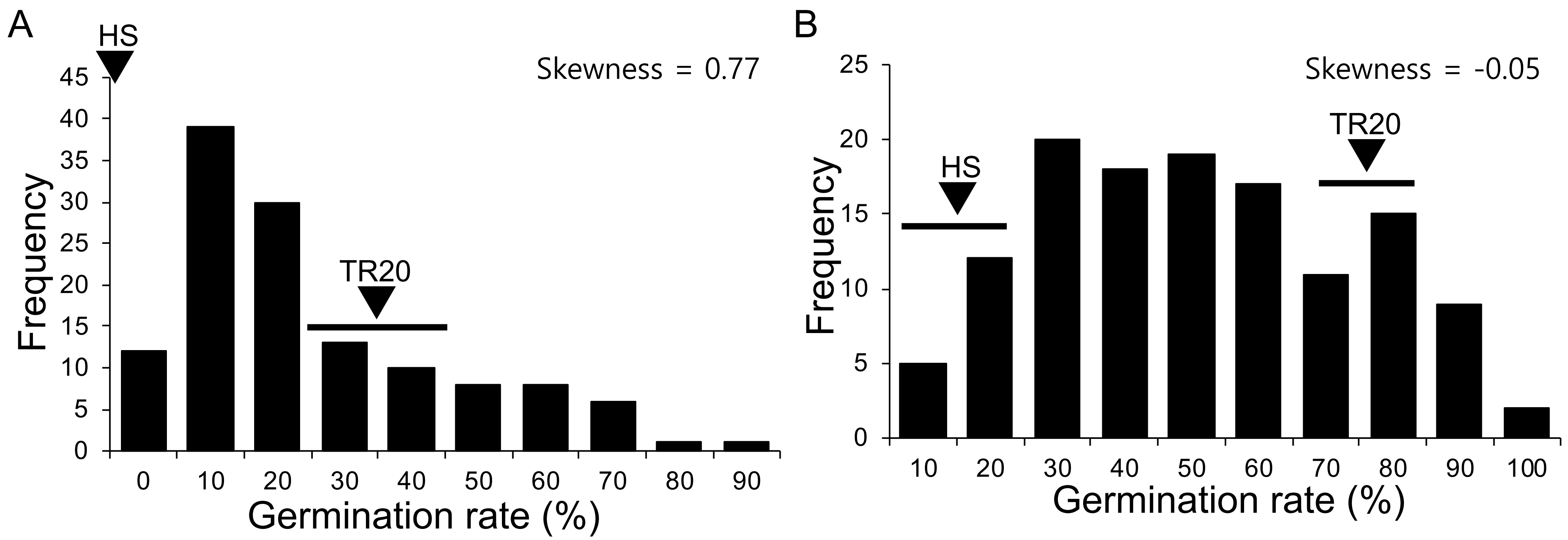

Figure S3. Frequency distribution of LTG at 6 days (A) and 7 days (B) after incubation of the 224 F_2 plants, respectively. Triangles and horizontal lines denote mean germination rates and standard error of the parental lines, respectively.

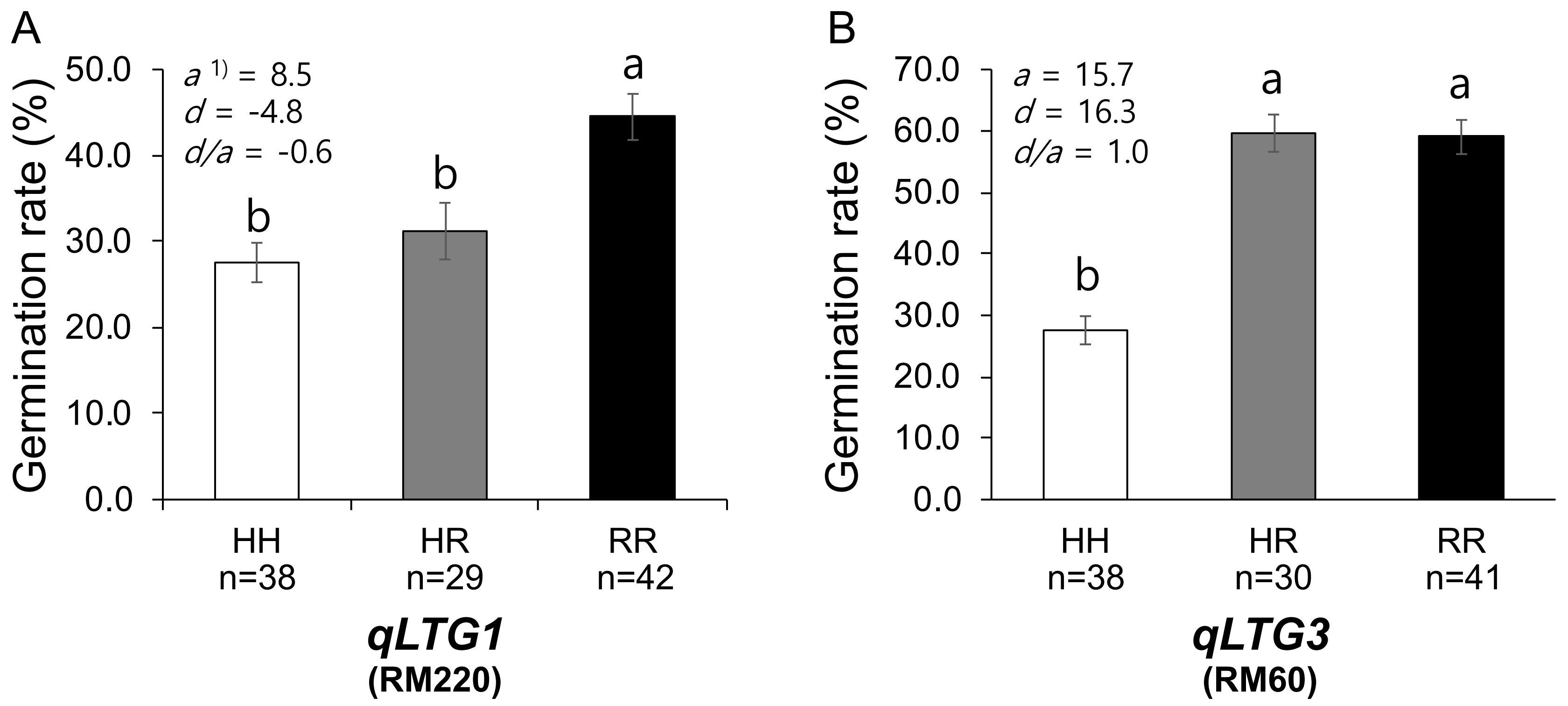

Figure S4. Comparison of the germination rates in plants that are segregating at (A) *qLTG1* and (B) *qLTG3* regions without other LTG QTL. RR, RH, and HH, mean *O. rufipogon* homozygous, heterozygous, and Hwaseong homozyg ous with the number of F_2 individuals, respectively. Additive effect (*a*) of the *O. rufipogon* allele, dominance effect of the *O. rufipogon* allele (*d*), and degree of dominance (*d/a*) were indicated in each figure. The same letter above box is not significantly different between the genotypes at *P* = 0.05 based on Tukey's test.

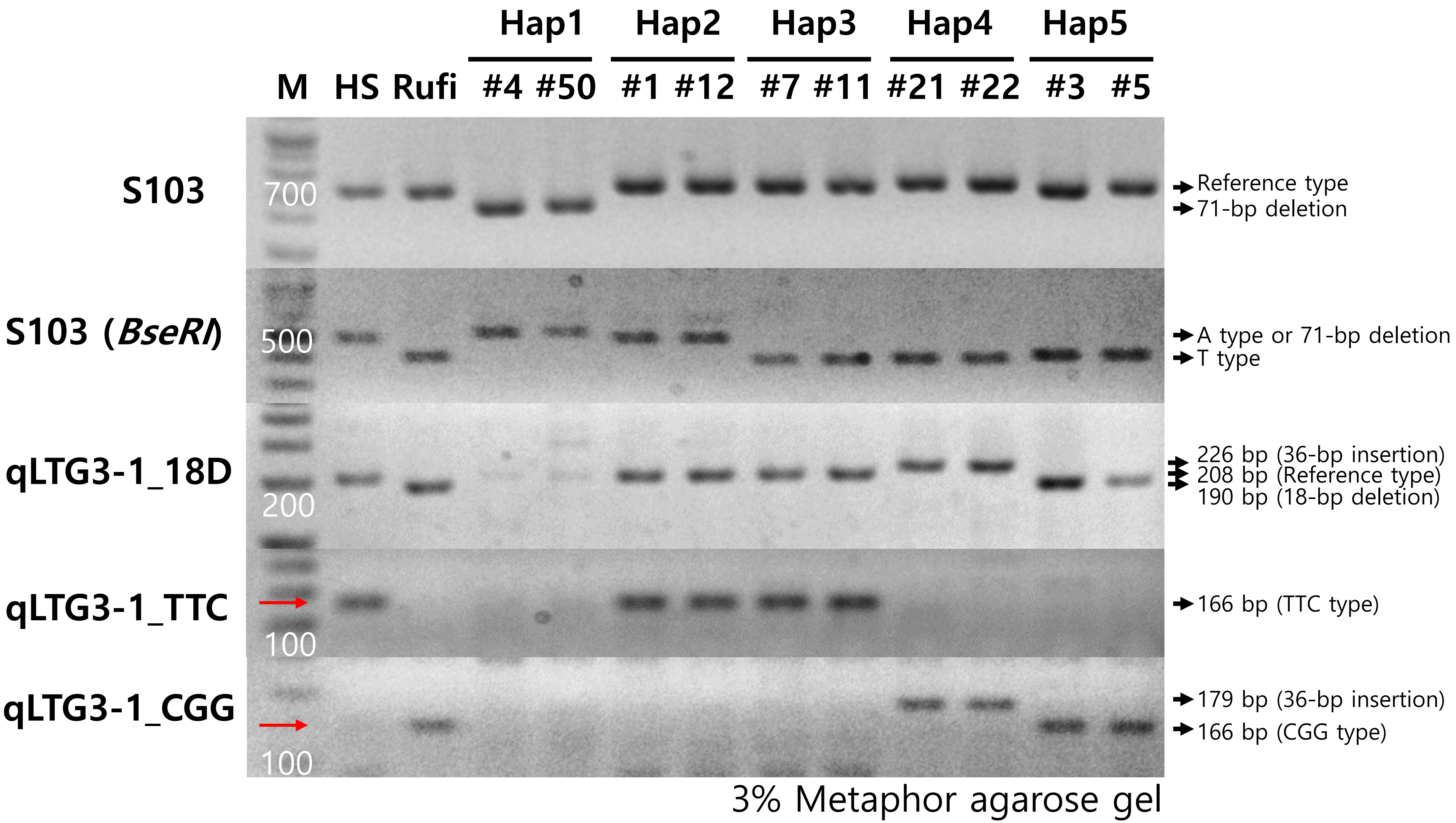

Figure S5. PCR amplicons of the markers used for genotyping parental lines and rice accessions for haplotype analysis. HS: Hwaseong, Rufi: *O. rufipogon*, #: RWG accession number, and M: 100-bp size marker.

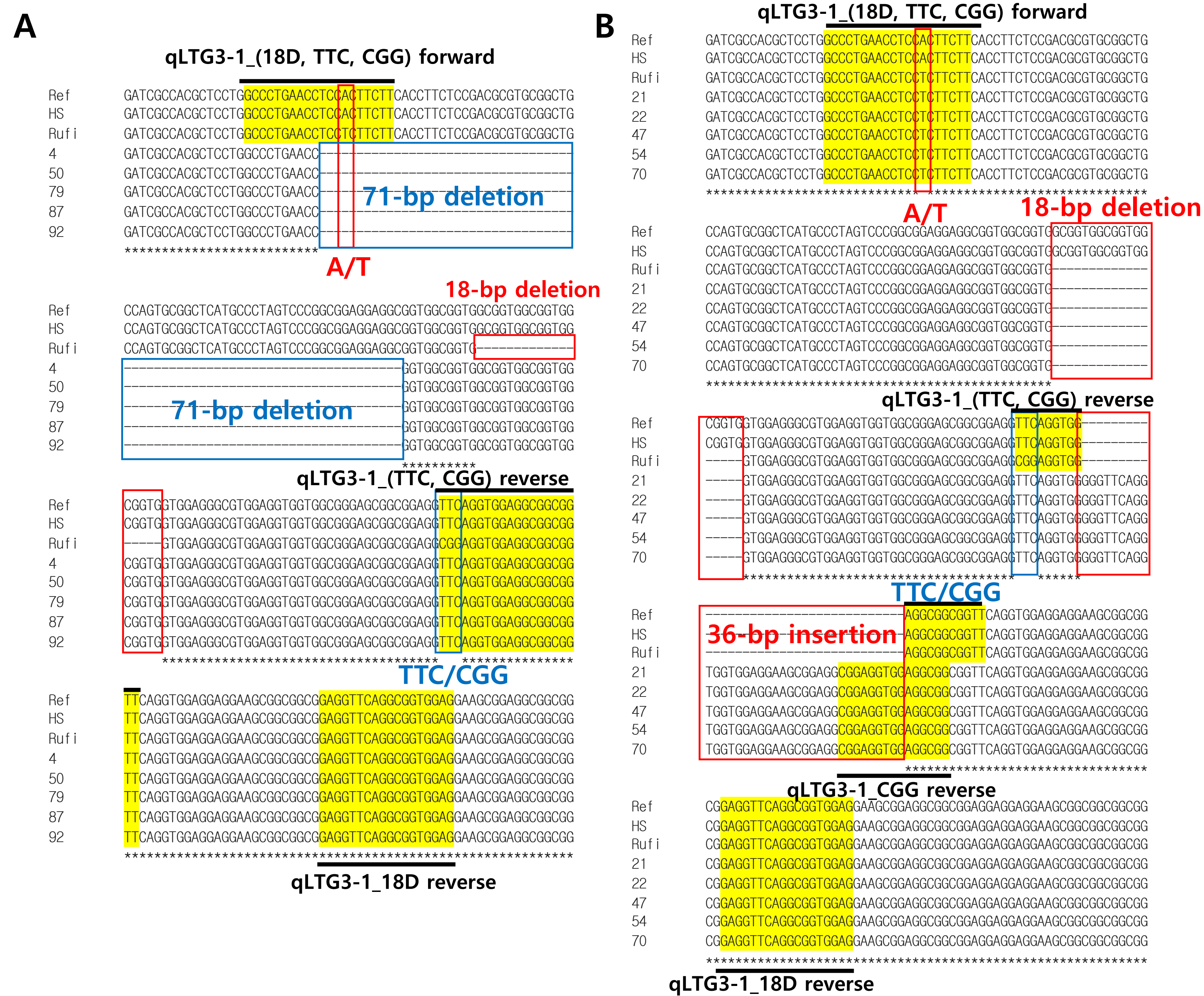

Figure S6. Sequence comparisons of the Ref (Nipponbare), HS (Hwaseong), Rufi (*O. rufipogon*) and (A) five rice accessions (RWG4, RWG50, RWG79, RWG87, RWG92) with a 71-bp deletion and (B) five rice accessions (RWG21, RWG22, RWG47, RWG54, RWG70) with a 36-bp insertion. Sequence and location of 3 markers are shown with an A/T SNP, 18-bp and 71-bp deletions, TTC/CGG, and 36-bp insertion variant site.
